# TeachBot: Towards teaching robotics fundamentals for human-robot collaboration at work

**DOI:** 10.1016/j.heliyon.2021.e07583

**Published:** 2021-07-19

**Authors:** Nicholas Stearns Selby, Jerry Ng, Glenda S. Stump, George Westerman, Claire Traweek, H. Harry Asada

**Affiliations:** a Massachusetts Institute of Technology (MIT), 77 Massachusetts Ave., Cambridge, MA 02139, United States of America; b Department of Electrical Engineering and Computer Science, MIT, United States of America; c Department of Mechanical Engineering, MIT, United States of America

**Keywords:** Adult learning, Human-computer interface, Human-robot interaction

## Abstract

The shortage of skilled workers who can use robots is a crucial issue hampering the growth of manufacturing industries. We present a new type of workforce training system, TeachBot, in which a robotic instructor delivers a series of interactive lectures using graphics and physical demonstration of its arm movements. Furthermore, the TeachBot allows learners to physically interact with the robot. This new human-computer interface, integrating oral and graphical instructions with motion demonstration and physical touch, enables to create engaging training materials. Effective learning takes place when the learner simultaneously interacts with an embodiment of new knowledge. We apply this “Learning by Touching” methodology to teach basic concepts, e.g. how a shaft encoder and feedback control work. In a pilot randomized control test with a small number of human subjects, we find suggestive evidence that Learning by Touching enhances learning effectiveness in this robotic context for adult learners. Students whose learning experience included touching the robot as opposed to watching it delivers the lessons showed gains in their ability to integrate knowledge about robotics. The “touching” group showed statistically significant gains in self-efficacy, which is an important antecedent to further learning and successful use of new technologies, as well as gains in knowledge about robotic concepts that trend toward significance.

## 1 Introduction

Amid the revolutionary changes to manufacturing and other industry sectors, the shortage of skilled workers who can use robots and advanced technologies are becoming a serious problem. A study by the Manufacturing Institute estimates that this shortage will leave two million unfilled manufacturing jobs in the United States alone [1]. The widening gap between required expertise and the skills of the available workforce is hampering technological growth in industries [2].

Workforce development for advanced robotics and automation is a crucial challenge. In the robotics and automation society, as well as in the education research sector, a number of valuable educational materials using robots have been developed. These include robotics programming instruments for elementary school children [3]; cellular-phone-based, low-cost robots for college students [4]; and industrial mobile robots for graduate students [5]. While education and training have traditionally targeted youth [6], the workforce shortage problem cannot be solved through youth education alone. It is necessary to reach out to a broader population including older generations, incumbent workers, and others lacking engineering education. The goal of this work is to develop a new methodology for reaching out to these broad populations, engaging them, and empowering them with the training and education necessary to become employed in jobs involving robotics and automation.

In the future, occupations in which employees work productively *alongside* robots will be ubiquitous. Decades of robotics research have given robots more intelligent behavior such as understanding human intentions and coordinating motion alongside humans [7], [8]. Collaborative robotic systems are growing rapidly in popularity in industry. In the past five years, the market for collaborative robots has more than doubled to over $1 billion [9]. Appropriately, surveys of workers in manufacturing industries have shown worker attitudes toward these collaborative robotic systems to be both nuanced and highly varied, with little clarity regarding of what robots are capable and how they can be used effectively in the workplace [10]. We believe that merely making robots “smarter” is not enough to yield a productive human-robot relationship. The humans must also learn, so that they are better prepared for interacting with robots.

An important consideration for teaching the knowledge necessary to utilize advanced robotics will be each individual's self-efficacy for interacting with robotics [11]. High levels of self-efficacy for completing work-related tasks have been linked to good service quality, efficiency, and effectiveness in the work place. In contrast, individuals with low levels of self-efficacy for tasks have difficulty completing them, despite being capable in terms of intellect or skill [12]. Furthermore, intrinsic interest in any activity, including robotics, may be cultivated by increasing self-efficacy in individuals with respect to that activity. Therefore, a robotics workforce education system should attempt to increase learners' self-efficacy for interacting with robots.

Sitting in a classroom, people are often unengaged, merely gaining superficial knowledge. Similarly, online learning, particularly large open courseware, has often failed to engage the broader population [13]. The question is: how can these individuals be better engaged, and thus achieve a more fruitful learning experience? Recent research in teaching science addresses what is missing in those learning methodologies. Specifically, investigations into the effectiveness of “hands-on” activities as tools for learning have concluded that a key factor to successful learning activities involves allowing learners to manipulate physical objects with their own hands. Modern neuropsychology research has indicated that these interactions with physical objects enhance students' learning and creates a solid “embodiment” of knowledge in their brains [14]. Robotics education lends itself well to a hands-on learning format. However, the exact benefits of embodied learning is still an open research question in learning sciences. Many physical human-robot interaction studies have demonstrated the effect of embodiment [15], [16], [17], [18], [19], [20], [21], [22]. We can utilize the advantages of object-mediated learning to engage trainees and enhance their learning experiences. As opposed to video or classroom lectures, interactive learning with a robot implicitly instructs its usage. An interactive robot is an intriguing “object” that can engage a broad population of learners.

**Figure 1:**
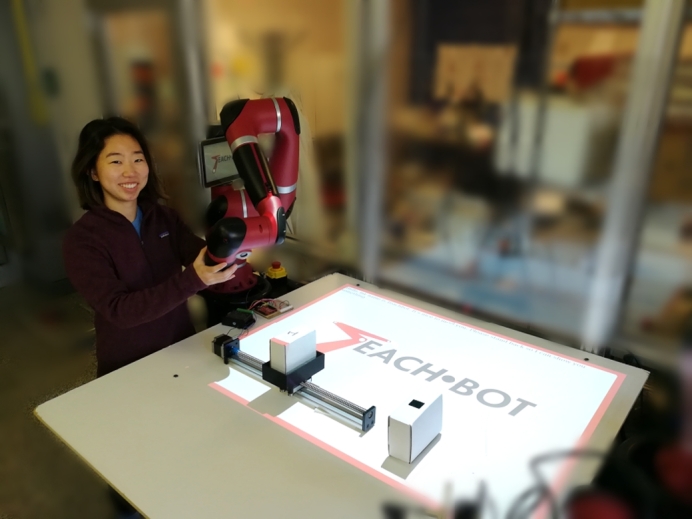
The TeachBot System. A collaborative robot, a cloud computer (not shown), a projector (not shown), and peripheral devices.

In the following sections, a novel workforce training system, called TeachBot, is introduced. Extending the object-mediated learning concept, we develop a new methodology for teaching the basics of robotics through physical interactions with the robot, referred to as *Learning by Touching*. We hypothesize that people will be more confident and comfortable working alongside robots if they have an opportunity to engage in Learning by Touching with a robotic instructor. The concept is implemented on a collaborative robot system connected to an online learning environment as shown in Fig. 1. We investigate the hypothesis that Learning by Touching helps learners become more comfortable with and confident about the material than if they learned the same concepts without physical interaction. We present results from randomized control trials indicating that this new platform significantly improves learner self-efficacy. Furthermore, an experiment investigating the effect of Learning by Touching on knowledge gained opens a promising future of development on this platform to upskill adult learners in collaborative manufacturing robotics.

## 2 Literature review

The scientific foundation of object-oriented learning can be found in brain and cognitive sciences. Recent neurological studies have revealed that *thinking about* completing a task and actual muscular motion are closely related. Indeed, imagining doing an activity and actually doing it excite the same parts of the brain [23], [24]. Hauk et al. [25] have also shown that the specific segments of the brain responsible for linguistic communication and those for manipulating tools are colocated over a wide range of the brain. For the growth and maturation of *homo sapiens*, this implies that the neurological development associated with physical actions and that of high-level language skills concurrently occurred [26].

In fact, the same areas of the brain responsible for motor control are also among the most capable of supporting learning and the emergence of skilled behavior. Strick et al. [27] observed that certain regions of the cerebellum are adaptively plastic, allowing them to modify internal models of systems so that complex task execution can be fast and accurate. Further, when a person is attempting to master a new skill, the same adaptively plastic regions continuously construct forward models predicting what sensory input will result from muscle motion. Then, much like a real-time adaptive control algorithm, the brain trains its understanding of reality by reversing the model and backpropagating the error. The human cerebellum learns by performing real-time adaptive control using the body's muscles as actuators and its sensory perception as feedback. The cerebellum is evolutionarily designed to excel at Learning by Touching.

Previous work has leveraged this phenomenon in a variety of applications. While treating patients who suffered brain damage, Doidge [28] discovered that playing language games designed to excite the adaptive learning centers of the cerebellum significantly improved patients' ability to re-learn motor skills. Robotic teachers have been used to help stroke patients recover through muscle therapy [29], [30], [31] and teach children diagnosed with autism important language skills by inviting them to play games designed to excite the cerebellum [32], [33], [34]. Furthermore, many successful consumer robots such as LEGO Mindstorms [35], Root [36], and Hapkit [37] educate K-12 and college students in robotics and automation. Many studies have concluded that such products are effective at teaching young students the fundamentals of robotics [38], [39], [40], [41], [42], [43], [44]. Further work by Troussas et al. [45], [46] has leveraged the ability of hands-on, interactive learning to implement fuzzy logic-based, automated curriculum personalization, demonstrating that even simple individualization of the learning environment can positively impact learners.

These findings imply that providing dual, concurrent stimuli to the learner, one physical and the other conceptual, will improve learning effectiveness. This is the key idea underpinning the TeachBot methodology.

## 3 TeachBot: a robot that teaches robotics to humans

The goal of the current work is to create a new workforce development methodology that can effectively engage broad populations, including older generations. Beyond merely providing educational materials for hands-on learning, we must invite and engage people who might otherwise be unable to get training. Our approach is to develop a new workforce training system that integrates oral explanation and guidance, graphics and animations that are coordinated with the vocal instruction, physical demonstration of the machine in a realistic setting, and concurrent body movements that create touch and proprioceptive sensations. Integrating these will allow us to create new curricula. We hypothesize that people will be more confident and comfortable working alongside robots if they have an opportunity to interact with a robotic instructor. Here, we propose an integrated, verbal-graphical-demonstrative-and-touchable system called TeachBot.

TeachBot is an autonomous, robotic instructor that introduces workers on a manufacturing line to robotics. TeachBot plays a dual role: an instructor delivering a lecture and a demonstration machine that can execute programmed movements and perform various tasks. It is the physical extension of an online course where lectures and laboratory sessions are seamlessly integrated. It requires no on-site human instructor; instead, trainees interact directly with TeachBot. Course materials are presented to produce a synergistic effect: integrating verbal and pictorial instructions into physical demonstrations and laboratory exercises. Learners will not only listen to instructions from the robot, but also participate in demonstrations with the robot. This methodology aligns with the latest learning science research on object-mediated learning and embodiment. TeachBot aims to attract a broad range of learners with diverse backgrounds.

Fig. 1 shows the overview of a TeachBot prototype system. It consists of:

(A) A robot that can interact with learners physically. The system is built with the technology of collaborative robotics that allows humans to safely interact with the robot;
(B) A computer that accesses a cloud-based learning platform, delivers instruction materials, and controls the entire system;
(C) An interactive projector that displays graphs, scripts, and other images for instruction and communication; and
(D) Peripheral devices and materials, including workpieces, jigs and fixtures, parts-feeders, and belt conveyors.

Instead of using a computer monitor, the projector is used for displaying various images on a large worktable. Learners around the worktable focus just on the robot and the worktable rather than distributing their attention to a computer monitor, keyboard, and other places. All information is communicated both verbally and visually by combining TeachBot with the projector. The robot is synchronized with the audiovisual system so that the explanation of concepts and techniques is seamlessly integrated and coordinated with the physical demonstrations. When talking about the three-dimensional orientation of an object, for example, TeachBot immediately demonstrates with its posture how to hold an object in the desired orientation. TeachBot also points to objects with its end effector and speaks with gestures. TeachBot can be programmed to speak any language.

**Figure 2:**
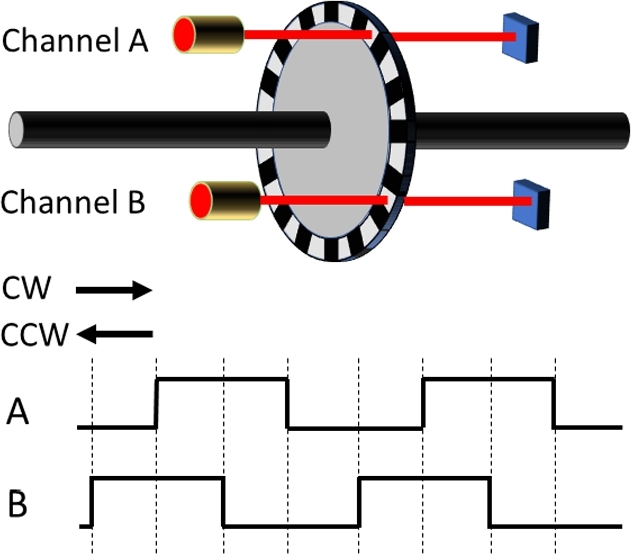
Example of Traditional Curriculum. Diagrams explaining the principle of an optical shaft encoder.

**Figure 3:**
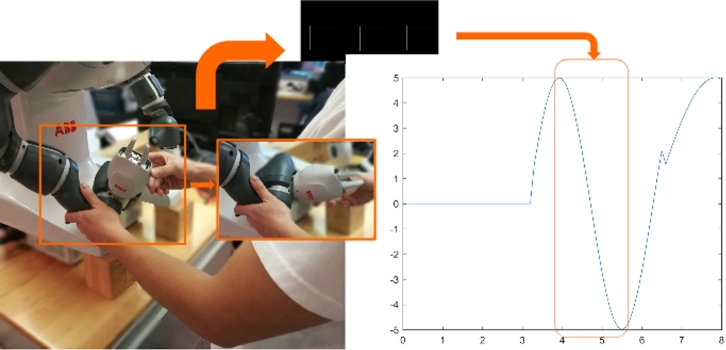
Learning by Touching. In contrast to traditional education, a learner touches the robot and rotates a specific joint. Simultaneously, the learner observes the shaft encoder of the joint ticking and indicating the joint angle on a display.

This unique integration of oral instruction, graphics, demonstration, and physical interaction will allow us to create unique curricula. Suppose that a learner with limited engineering background is to study how a robot can precisely move its joints to desired angles. A shaft encoder plays a key role in closed-loop control by measuring joint angle. The principle of a shaft encoder, however, is challenging to understand for most people. A diagram explaining the principle of an optical shaft encoder is confusing (see Fig. 2). Allowing the learner to touch and move a joint of the TeachBot with their own hands while they observe changes in the signal from a shaft encoder enables more effective learning (see Fig. 3).

As they push the robot arm and observe the effects of their actions resulting in the generation of signals, learners make a mental connection between the motion and measured signals. Their muscular action and visual observation occur concurrently, creating a synergistic “embodiment” of the function of the shaft encoder. This is not merely a standard hands-on laboratory experience. No complex reasoning is required. The learner can understand intuitively what a shaft encoder can do by touching and moving it: Learning by Touching.

**Figure 4:**
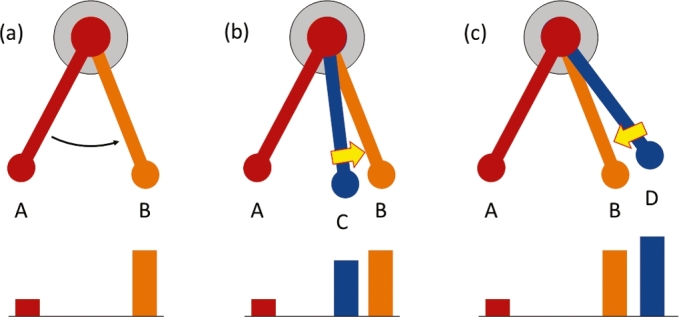
Encoder Readings. (a) TeachBot attempts to move its arm from point A to point B. (b) TeachBot undershoots, stopping instead at point C. (c) TeachBot overshoots, stopping instead at point D.

“Feedback control,” a basic technique for controlling robots and all kinds of machines, is not easy to understand for the majority of people who have a limited engineering background. Even engineering students have difficulty in understanding the concept [37]. TeachBot takes a different approach to teaching the principle of feedback control to non-engineering people. A learner experiences feedback through manual execution of actions to correct TeachBot's joint position. Suppose that TeachBot attempts to move from point A to point B as shown in Fig. 4(a). With no feedback, the TeachBot may not reach point B. Instead it may stop at point C, as shown in Fig. 4(b). Next, TeachBot asks the learner to move the arm towards the destination, point B. The learner pushes the arm to the right. Then, TeachBot tries again, but this time overshoots to point D, as shown in Fig. 4(c). The learner again pushes the arm back towards the destination. These are “manual” feedback operations completed in a primitive manner. TeachBot explains that the actions the learner has taken can be generated by comparing the encoder reading at the current position against the one at the destination. For example, if the current reading is smaller than the destination reading, move right. If it is larger, move left. This is a demonstration of feedback control that a broad range of people can understand. Experiencing manual feedback where physical actions and visual observations take place simultaneously can help the learner understand the principle.

## 4 Materials and methods

### 4.1 Implementation

We implement TeachBot on a Rethink Robotics Sawyer robot arm [47]. The Sawyer is connected to a Dell Precision 7720 laptop running Ubuntu 18.04. The Sawyer is programmed in Python on ROS Melodic using Rethink Robotics' Intera SDK [48].

**Figure 5:**
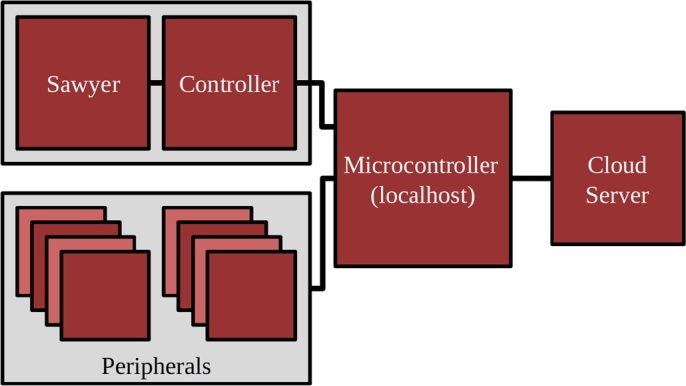
Software Architecture. A cloud-based server written in Node.JS communicates through a local browser-based client to the robot using ROS.

The software architecture is illustrated in Fig. 5 and released under a BSD-3-Clause open source software license at [49]. All commands to the robot are coordinated by a web client written in HTML, CSS, and JavaScript and running on Firefox. The web client communicates with a cloud-based server written using Node.JS. The server acts as the master node, issuing commands through the client to the robot to move and requesting joint states. The server also commands the browser to display graphics and animations and play audio.

TeachBot graphics and text are projected onto a worktable by an Epson Powerlite projector. The table was built in-house out of aluminum and melamine board to optimize the projection surface.

The concepts taught by TeachBot in this study are categorized into seven sub-modules: Motors and Degrees-of-Freedom, Encoders, Feedback, Kinematics, Memory, Orientation and Position, and Waypoints.

### 4.2 Framework and methodology

We conducted a pilot human subject experiment based on a protocol approved by the Massachusetts Institute of Technology Committee on the Use of Humans as Experimental Subjects (COUHES), #1806389401. The objective was to investigate the efficacy of robot-mediated learning.

In particular, this experiment focuses on the evaluation of the TeachBot curriculum along two axes: self-efficacy and knowledge gained by the learner. In addition to quantifying the knowledge gained from the curriculum, we include an evaluation of self-efficacy because previous studies have indicated the importance of self-efficacy for learning and applying knowledge on the job [11], [12]. We based our self-efficacy testing materials on surveys from that literature.

More than simply investigating TeachBot's ability to upskill workers, this pilot study specifically attempts to evaluate the Learning by Touching methodology against online video learning. For this reason, we selected a methodology of randomized control trials in which the control group took a video-based version of the TeachBot course. In order to isolate the effect of physical interaction with TeachBot, we designed the control group's video curriculum to include exactly the same audiovisual content as the experimental group's curriculum: namely, a video recording of an example student taking the TeachBot course.

#### 4.2.1 Participants

**Figure 6:**
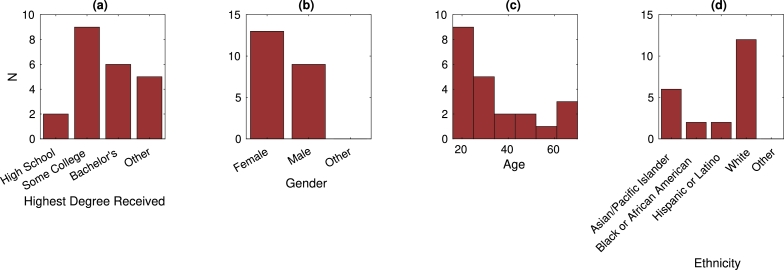
User Study. (a) Number of subjects with differing levels of education. (b) Number of subjects of each gender. (c) Histogram of subject age. (d) Number of subjects of each ethnicity.

Subjects, like the workforce they represented, were diverse: men, women, young adults, retirees, people who worked in jobs with robots, and people who were uncomfortable with the idea of robots in the workplace. Twenty-two subjects were recruited from Central Square in Cambridge, MA, USA and pre-interviewed to ensure none had a four-year degree in STEM. Before the TeachBot course, subjects were given a short survey to gather demographic information (see Appendix A) and the results are illustrated in Fig. 6. The plurality of subjects had some education beyond high school not including attaining a bachelor's degree. There were slightly more women in the sample than men. Ages ranged from 18 to 68 years old. Participant ethnicity was varied. Participants were given a $25 gift card and a certificate of completion for their participation in the experiment.

#### 4.2.2 Design

The testing of each subject was completed in three parts, with the whole experiment taking up to 90 minutes per participant:

1. **Pre-Test:** Subjects were evaluated for baseline knowledge and self-efficacy.
2. **Learning Module:** Subjects completed either the TeachBot or video course.
3. **Post-Test:** Subjects were evaluated again for newly learned knowledge and changed self-efficacy.

#### 4.2.3 Tests

The pre- and post-tests each had 34 questions. Of these, six measured subject self-efficacy by asking subjects to rate how confident they would be completing various tasks involving a robot. The remaining 28 questions evaluated subjects' knowledge of robotic systems. The 28 knowledge-evaluation questions were divided evenly among the seven concepts taught by TeachBot discussed in Sec. 4.1. Knowledge regarding each concept was tested using four questions:

• One **verbatim** question generated from ideas and information explicitly stated in the learning module and that required subjects to merely recall the correct responses. These questions required the shallowest level of understanding for the subject to answer correctly. For example, “Consider a robot with position feedback control. You push the arm away from its target position, then let go. What does the arm do?”
• One **integration** question also generated from ideas and information explicitly stated in the learning module, but which required subjects to integrate two or more ideas from the learning module. Thus, integration questions required a slightly deeper level of understanding. For example, “What devices allow a robot arm to change its position and orientation?”
• Two **inference** questions that required subjects to generate ideas beyond the information presented in the learning modules, thus requiring the deepest level of understanding. For example, “The motors in this activity can only rotate. How can you move motors to make something move in a straight line?”

The three categories of questions represent different levels of cognitive activity required to respond to the question; these categories were also considered to be indicative of question difficulty [50], [51]. Additionally, all subjects were given a five-question survey requesting their level of education, gender, age, occupation, and ethnicity. The results of this demographic survey are shown in Fig. 6.

After completing the learning module, subjects took a post-test to evaluate self-efficacy for knowledge gains that could be attributed to completion of the learning module. The post-test was identical to the pre-test except that it did not include the demographic survey and the question order was shuffled.

Please see Appendix A for the complete test materials.

#### 4.2.4 Experimental and control groups

**Figure 7:**
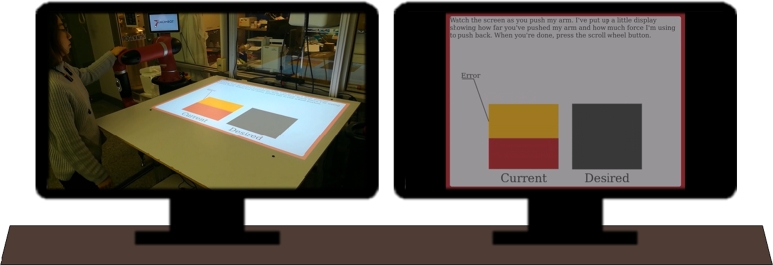
Control Group Video Learning Module. Two adjacent displays synchronized to present equivalent content without Learning by Touching to subjects in the control group.

Before beginning the pre-test, subjects were asked to draw a piece of paper randomly from a table. Half of the papers directed the subject into the experimental group, half into the control group. The papers were discarded after each draw to guarantee an equal number in each group. The experimental group participated in an approximately 20-minute, interactive, hands-on learning module conducted by the TeachBot. The control group was told to watch a video on two monitors showing a model subject taking the hands-on TeachBot course. As illustrated in Fig. 7, one monitor showed what the TeachBot projected onto the table while the other showed a video of the model subject interacting with the TeachBot. The videos were synchronized with each other and a single audio stream to teach content equivalent to what the experimental group received but without Learning by Touching. The videos can be found at the following links: [52], [53], [54], [55]. Since the topical content of the lesson is identical for the treatment and control groups, this research design allows us to assess the extent to which touching the robot during the lesson, as opposed to simply seeing the lesson, is associated with higher learning outcomes.

### 4.3 Evaluation

Similar to VanLehn's evaluation [56] quantifying student learning, we measured differences in subjects' scores on the pre- and post-tests. For each learner, two metrics were calculated: a self-efficacy gain to measure a growth in subject confidence with robotics between the pre- and post-tests, and a learning gain to determine how much a subject learned between taking the tests.

#### 4.3.1 Self-efficacy gain, G

On both the pre- and post-tests, subjects were asked to rate how confident they would be completing each of six unique tasks involving a robot on a scale from one to ten with one being least confident and ten being most confident. These ratings were assigned a label $X_{i,j}$ where *i* is the question number and *j* is the test identity: pre-test or post-test:

(1) $$X_{i,j}\in [1.\;.10]$$

Next, the differences between each subject's $X_{i,j}$'s were calculated across the pre- and post-tests:

(2) $$\Delta_{i}=X_{i,\mathrm{post}}-X_{i,\mathrm{pre}}$$

Therefore, $\Delta_{i}$ is used to symbolize the amount of confidence gained by a subject during the learning module.

Some subjects began with a greater initial level of self-efficacy for robotics than others. Simply comparing the Δ's between the control and experimental groups weights subjects with less self-efficacy more heavily because such subjects have more potential to improve their scores between tests. To account for this effect, we normalized each Δ by computing a ratio of the self-efficacy gained to the amount the subject could have gained:

(3) $$G=\frac{\sum_{i}\Delta_{i}}{10N_{G}-\sum_{i}X_{i,\text{pre}}}$$

where $N_{G}=6$, the number of self-efficacy questions. $N_{G}$ is multiplied by 10 because the maximum rating for each of these questions is 10. Simply put, G is the self-efficacy gained by a subject normalized by the amount they could have gained when they began the experiment, the “self-efficacy gain”, with positive values indicating higher self-efficacy and negative values indicating self-efficacy lost.

#### 4.3.2 Learning gain, Λ

To quantify learning, we used subjects scores on the 28 knowledge-evaluation questions. Subjects answer to these questions on the pre- and post-tests were assigned a binary grade, $X_{i,j}$ where *i* is the question number and *j* is the test identity: pre-test or post-test:

(4) $$X_{i,j}=\left\{ \begin{array}{c} 1,\text{if correct}\\ 0,\text{otherwise} \end{array}\right.$$

Similar to the calculation of self-efficacy gain above, Δ's were computed for each of the 28 knowledge-evaluation questions using Eq. (2). For these questions, $\Delta_{i}$ is used to symbolize the quantity learned by a subject between taking the pre- and post-tests, with positive values indicating new knowledge learned and negative values indicating knowledge forgotten or mislearned.

Some subjects came in with significantly more understanding of the field of robotics and automation than others. As in the above calculation of self-efficacy gain, simply comparing the Δ's between the control and experimental groups weights subjects with less prior knowledge more heavily because such subjects have more potential to improve their scores between tests. To account for this effect, we normalized each Δ by computing a ratio of the amount learned to the amount the subject could have learned:

(5) $$\Lambda =\frac{\sum_{i}\Delta_{i}}{N_{\Lambda }-\sum_{i}X_{i,\text{pre}}}$$

where $N_{\Lambda }=28$, the number of knowledge-evaluation questions. Simply put, Λ is the amount learned by a subject normalized by the amount they did not yet know when they began the experiment, the learning gain.

#### 4.3.3 Analysis by question type

The learning gain, Λ, was also computed for each category of question using Eq. (5). Separating results into verbatim, integration, and inference categories revealed the areas in which TeachBot was the most helpful and areas that could be improved during future iterations of the project. Identifying TeachBot's strengths over traditional methods verifies the potential utility of the system and ensures that no critical gaps exist.

## 5 Results and discussion

**Figure 8:**
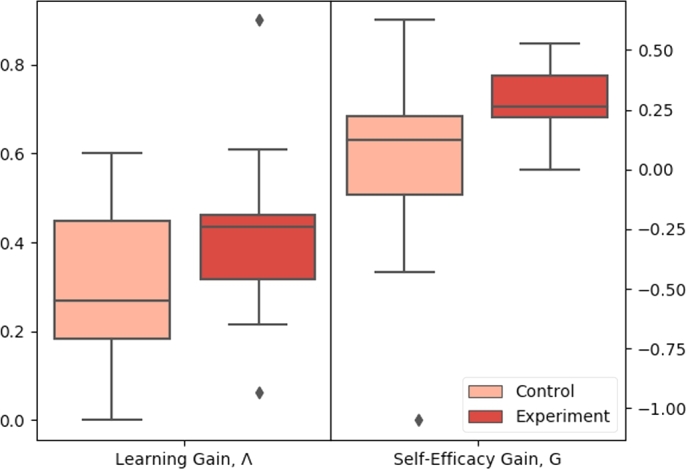
Comparison of performance of the control and experimental groups. (a) compares box plots of the self-efficacy gain, G, demonstrating that subjects in the experimental group tended to experience a greater gain in self-efficacy than those in the control group. (b) compares box plots of the learning gain, Λ, demonstrating that subjects in the experimental group tended to learn more than subjects in the control group.

We evaluate how well TeachBot improves subjects' understanding of fundamental robotics concepts, as well as their self-efficacy regarding those concepts. We use a one-tailed, two-sample, unpaired, heteroscedastic *t*-test to compare the results of both metrics across groups. For each metric, this test allowed us to test the hypothesis that the mean learning or self-efficacy gain of a learner who takes the TeachBot course is greater than that of a learner who watches a video lecture containing the same information. Values of the learning gain, Λ, and self-efficacy gain, G, were computed for each subject. We present the results in Fig. 8 and Table 1 and observe the following:

• **Subjects in the TeachBot course gained more self-efficacy for interacting with robots than those in the video course.** As shown in Fig. 8 and Table 1, the median G for the experimental group is 0.26 compared to 0.13 for the control group, implying that subjects who took the TeachBot learning module gained significantly more confidence with robots than those who took the video course. The results from the *t*-test allow us to reject the null hypothesis with p<0.05. Physically interacting with the TeachBot system increased learner confidence in and comfort with their understanding of the material.
• **Subjects in the TeachBot course learned more than those in the video course.** As shown in Fig. 8 and Table 1, all 11 of the subjects in the experimental group performed better on the post-test than they did on the pre-test. The median Λ for the experimental group is 0.43, implying that the TeachBot learning module taught the median learner 43% of the total amount they could have learned. The median Λ for the experimental group is 0.27. The results from the *t*-test allow us to reject the null hypothesis with p<0.2. We also experimented with a more complex grading rubric, replacing the binary grading scheme in Eq. (4) with ternary grades and effectively offering partial credit. The effect of this was to increase everyone's scores and eliminate the learning differences between groups.

**Table 1:** Results for Self-Efficacy and Learning Gains. Pairwise Comparisons Using a One-Tailed, Two-Sample, Unpaired, Heteroscedastic *t*-Test.

Metric	Mean difference	*p*-value
Self-Efficacy Gain, G	0.264	0.046**
Learning Gain, Λ	0.106	0.120*

* Significant with α=0.2. ** Significant with α=0.05.

**Figure 9:**
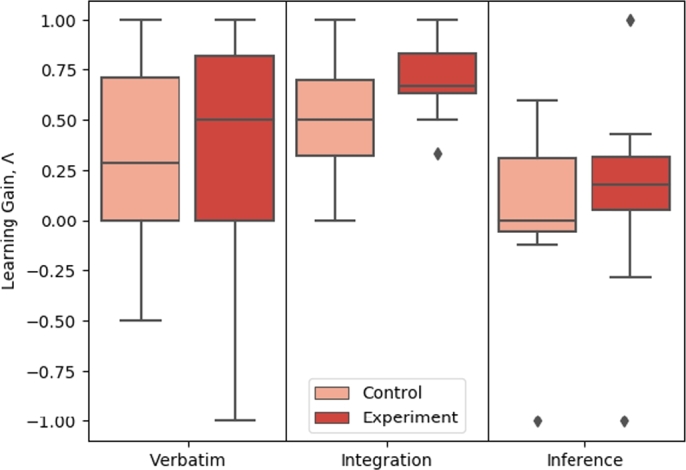
Comparison of performance of subjects between different categories within the control and experimental groups. While members who took the TeachBot course (the experimental group) scored higher across all three categories than members who took the online video lecture (the control group), the effect size and significance varied distinctly between question category.

Additionally, analyzing the question category learning gains highlights the strengths of the TeachBot curriculum as well as specific areas for improvement. Subjects in the TeachBot course did better on integration questions than those in the video course. As shown in Fig. 9 and Table 2, subjects in the experimental group produced an integration-specific learning gain 0.158 higher than those in the control group. Results from the *t*-test allow us to reject the null hypothesis with p<0.2. Physically interacting with embodiments of the concepts being learned allowed users to better understand connections between those concepts. Previous work has found that hands-on learning environments must have highly integrated curricula in order to help learners grasp connections between ideas [57]. The structure of robotics learning is inherently connected: in order to understand waypoints, one must understand feedback control, and in order to understand feedback control, one must understand encoder functionality. Enabling learners to touch and interact with a robot through demonstrations of knowledge in sequence encourages the formation of cognitive connections between related concepts.

**Table 2:** Results for Question Category Learning Gains. Pairwise Comparisons Using a One-Tailed, Two-Sample, Unpaired, Heteroscedastic *t*-Test.

Question category	Mean difference	*p*-value
Verbatim	0.036	0.436
Integration	0.158	0.136*
Inference	0.111	0.226

* Significant with α=0.2. ** Significant with α=0.05.

However, similar improvement was not seen in subjects' ability to infer new knowledge about robotic systems. We hypothesize that this is because, even though the TeachBot module was successful in guiding learners to develop a robust understanding of how concepts in automation and system integration build upon each other, we did not design enough inference-related material into the module. The learning modules we designed focused on helping learners connect new concepts to ones they already mastered, but did not guide learners to infer new knowledge about robot systems. Clearly, this is an area for improvement in future iterations of the curriculum.

As shown in Fig. 9 and Table 2, subjects in the TeachBot course also did not show a significant difference in recalling verbatim information as subjects in the video course. Video learning is likely equivalent to the TeachBot system in terms of rote memorization. This is likely because physical interaction only helps construct a more robust mental model of the robot system. A learner will not be significantly more likely to recall a fact if it is spoken by a robot in front of them than if it is spoken by a robot in a video. This result is in line with previous work on the impact of hands-on learning on immediate recall [58].

**Figure 10:**
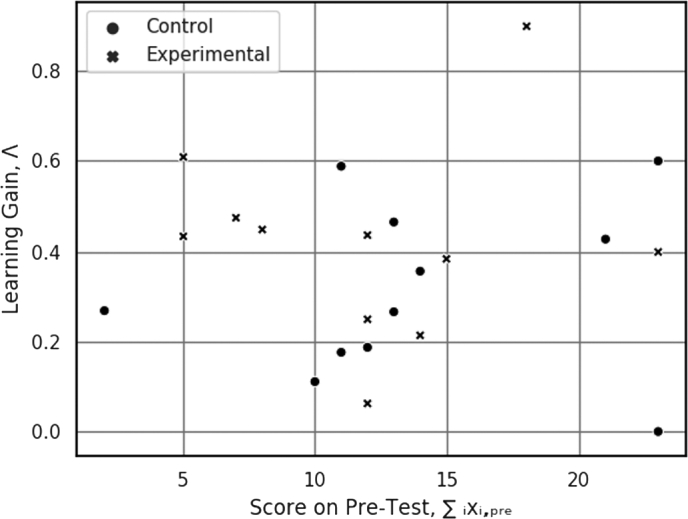
Comparison of subjects' pre-evaluation scores with their learning gain. Subjects who had more robotics knowledge initially did not experience a higher learning gain. The *r*-value for the control group was 0.134 and the *r*-value for the experimental group was 0.026.

Finally, there is no correlation between pre-test score and learning gain. As illustrated in Fig. 10, the learner's score on the pre-test is not predictive of their learning gain. Comparing pre-evaluation scores and learning gains reveals no significant correlation between initial scores and Λ. This indicates that the normalization in Eq. (5) successfully removed the effect of different initial knowledge state from the analysis.

These results are promising indicators that the TeachBot system would be a powerful tool to upskill adult workers to be confident and comfortable working with collaborative manufacturing robotics. This small pilot experiment indicated self efficacy gains for subjects who took the TeachBot course statistically significantly greater than subjects who completed a purely video-based curriculum. Learning gains for those who took the TeachBot course tended toward significance. Additionally, these results also help identify the specific strengths and weaknesses of the existing curriculum. Learners who took the TeachBot course saw gains in their ability to integrate knowledge across a variety of concepts, but more work needs to be done to enable students to infer new insights without being explicitly told. More generally, this experiment offers promising evidence toward the future of collaborative robots in education.

## 6 Conclusion

We have presented TeachBot, an automatic, robotic education and training system. TeachBot leverages the state-of-the-art in robotics, education, and neuropsychology to create an engaging learning environment and empower a diverse workforce with the skills necessary to contribute in advanced manufacturing occupations. A novel methodology has been developed by streamlining instruction, demonstration, and physical interaction with the robot. Human subject tests have been conducted to evaluate the efficacy of the new methodology, Learning by Touching. These human subject tests demonstrated that TeachBot provides a significant benefit to learners' self-efficacy for interacting with robots, their belief in their innate ability to interact productively with a collaborative robotic system. The findings about self-efficacy are important because self-efficacy is an important determinant of additional learning and effective use of new technologies. Even if there were no gain to “learning by touching” on conceptual measures, the fact that touch may improve self-efficacy makes it an important improvement, especially for non-traditional learners.

The findings of this pilot experiment have great implications for lifelong education and closing the manufacturing skills gap. We have proposed a novel educational platform, TeachBot, to upskill manufacturing workers to integrate, maintain, and operate collaborative manufacturing robotics in their places of work. We have provided the software to deploy TeachBot with an open source license. Preliminary human subject testing has demonstrated that learners who take the TeachBot course gain significantly more self-efficacy for manufacturing robotics than do learners who take a video version of the course. Measurements of knowledge gained by subjects in the experimental group also show an increase in learning that tends toward significance. These findings both motivate future development and study of the Learning by Touching methodology for collaborative manufacturing robotics education and offer insights into how the curriculum can be improved.

These findings build on previous studies investigating the efficacy of hands-on robotics in education [38], [39], [40], [41], [42], [43], [44]. Much of the prior literature in this space uses uncontrolled studies and focuses on K-12 or university-level robotics education. This pilot experiment focuses on adult learners, a relatively understudied population. It leverages a more rigorous randomized control trial research methodology to compare TeachBot against an alternative teaching method, video lectures, that contain the same content but lack the ability to facilitate Learning by Touching.

The concepts taught in this pilot experiment are basic, and the experiments conducted are preliminary. This will be expanded to teach a broader range of concepts and skills, and the experiment will be extended to more subjects. Currently, the research team is developing more course materials including trajectory generation and use of various sensors and vision systems. In addition to more material, the team is also developing more challenging activities that are directly applicable to real life scenarios found in manufacturing such as tasks involving pick and place and operating alongside CNC machinery. We envision a multiple-day training curriculum to teach learners a broad range of techniques that they need to know to begin working in advanced manufacturing.

While the focus of this pilot experiment is on self-efficacy and foundational knowledge gains, it is also important to investigate how taking the TeachBot course might additionally affect subjects' practical abilities to work with a robot on real manufacturing tasks. Additionally, future work should also include a qualitative component to address questions related to learners' relationship with the robot.

## Declarations

### Author contribution statement

Nicholas Stearns Selby, Jerry Ng: Conceived and designed the experiments; Performed the experiments; Analyzed and interpreted the data; Wrote the paper.

Glenda S. Stump, George Westerman, Claire Traweek: Analyzed and interpreted the data.

H. Harry Asada: Conceived and designed the experiments; Analyzed and interpreted the data; Contributed reagents, materials, analysis tools or data; Wrote the paper.

### Funding statement

This work was supported by the Advanced Robotics for Manufacturing Institute and the Massachusetts Manufacturing Innovation Initiative

### Data availability statement

Data will be made available on request.

### Declaration of interests statement

The authors declare no conflict of interest.

### Additional information

Supplementary content related to this article has been published online at https://doi.org/10.1016/j.heliyon.2021.e07583.

## Appendix A Supplementary material

TeachBot_Appendix.pdf This is the test administered to all participants before and after the learning module. With the exception of the Background Survey, which was only administered before the learning module, all questions were shuffled before being readministered after the learning module. TeachBot_Appendix.pdf
