# Supplementary material for: TeachBot: Towards teaching robotics fundamentals for human-robot collaboration at work
Source: Heliyon. 2021 Jul 19;7(7):e07583. doi: 10.1016/j.heliyon.2021.e07583 (PMC8322294; doi:10.1016/j.heliyon.2021.e07583)
Supplement: TeachBot_Appendix.pdf — This is the test administered to all participants before and after the learning module. With the exception of the Background Survey, which was only administered before the learning module, all questions were shuffled before being readministered after the learning module. [file mmc1.pdf]

Appendix A. Test Materials

This is the test administered to all participants before and after the learning module. With the exception of the Background Survey, which was only administered before the learning module, all questions were shuffled before being readministered after the learning module.

Appendix A.1. Background Survey

What is an email we can contact you at?

What is the highest degree or level of school you have completed? If currently enrolled, highest degree received.

- No schooling completed
- Nursery school to 8th grade
- Some high school, no diploma
- High school graduate, diploma or the equivalent (for example: GED)
- Some college credit, no degree
- Trade/technical/vocational training
- Associate degree
- Bachelor's degree
- Master's degree
- Professional degree
- Doctorate degree

Please specify your gender.

Please specify your age.

Please specify your occupation.

Please specify your ethnicity. Circle all that apply.

- White
- Hispanic or Latino

- Black or African American
- Native American or American Indian
- Asian / Pacific Islander
- Other

Appendix A.2. Self-Efficacy Survey

On a scale from 1-10 with 1 being least comfortable and 10 being most comfortable, how comfortable would you feel with completing the following:

- Interacting with a robot
- Working with a robot in the workplace
- Using a robot to help you assemble furniture
- Pursuing a career in robotics
- Explaining how a robot moves
- Learning how to program a robot

Appendix A.3. Knowledge Test

The twenty-eight questions designed to test conceptual knowledge about robotic systems represented seven topics. Each topic had one *ver batim* question, one integration question, and two inference questions. The questions were shuffled separately for the pre- and post-tests. The questions were as follows. The italicized text was not included.

1. Motors
   (a) *Ver Batim.* How many motors are used in this motion? [1] *Answer: 2*
   (b) *Integration.* How many motors do you need to move a robot arm along a line? *Answer: 2*
   (c) *Inference.* The motors in this activity can only rotate. How can you move motors to make something move in a line? *Answer: 2*

(d) *Inference.* How many degrees of freedom does your arm have, not including your hand? List them. *Answer: 7. Three shoulder degrees of freedom, one elbow, and three wrist.*

2. Encoders

   (a) *Ver Batim.* In a robot arm with three motors, at least how many encoders will there be? *Answer: 3*

   (b) *Integration.* What devices are needed for a robot to perform feedback control? *Answer: Motors and encoders.*

   (c) *Inference.* On what kind of motorized device wouldn't you need an encoder? Why wouldn't you need an encoder with a motor? *Answer: Many possible answers. One example could be a conveyor belt, because a conveyor belt often does not need to track it's current position.*

   (d) *Inference.* What in our bodies serves the role of encoders for the joints and muscles in our arms? *Answer: Proprioception or vision.*

3. Feedback

   (a) *Ver Batim.* Consider a robot with position feedback control. You push the arm away from its target position, then let go. What does the arm do? *Answer: Return to it's target position.*

   (b) *Integration.* Consider an arm with position feedback control. As you push the arm further from its target position, which of the following does it feel like most in terms of reaction force? (a) Pushing/pulling on a spring, (b) Dragging an oar through water, or (c) Lifting a heavy weight? *Answer: A.*

   (c) *Inference.* If one of the encoders broke such that, regardless of how the arm was positioned, it would always measure the same value, how would the arm react under feedback control? *Answer: The arm would drift.*

   (d) *Inference.* Consider a robot holding a large, heavy part and you need to tighten some screws on that part. If the screws are positioned all over the part in various orientations, how do I want my feedback controller to perform? *Answer: Stiff in position control, but compliant in orientation.*

4. Kinematics

   (a) *Ver Batim.* Look at the image of the robot. If it can only move the motor highlighted in red at the robot's center (the "shoulder" motor), draw the path along which it can move. *Answer: A circular path centered at the joint with radius equal to the arm length.*

   (b) *Integration.* For a robot with only two motors, draw a path from point A to point B in three steps? *Answer: A three-step path that obeys kinematics (i.e. only arcs centered at the two joints).*

   (c) *Inference.* If I have only one motor, what is the difference between moving from one point to another using one step versus using many steps? *Answer: More steps, nothing, or time.*

   (d) *Inference.* At least how many motors do I need in order to perform a standard pick and place operation? *Answer: 2 or 3.*

5. Memory

   (a) *Ver Batim.* Which of the following can be stored in memory before a robot begins a task? (A) Position; (B) Sequence of commands; (C) How the motors will need to react to a person pushing on the robot arm; (D) Why a collision will occur. *Answer: A and B.*

   (b) *Integration.* When I save a location in my memory, from which device am I getting that information? *Answer: Encoder.*

   (c) *Inference.* If I want my robot to move an object of interest around a square, how many unique points will I need to save in the robot's memory? *Answer: 4 or 5, considering starting and ending above the square.*

   (d) *Inference.* If I have to move a part over a box to a conveyor belt, what are the pros and cons each path? Path 1: two waypoints, forming a triangular path. Path 2: three waypoints, forming a trapezoidal path. *Answer: Discussion of efficiency and total distance traveled.*

6. Orientation/Position

(a) *Ver Batim.* True or False: Orientation is important in the execution of: (A) Picking up an object. (B) Placing an object into a container. *Answer: (A) True; (B) True.*

(b) *Integration.* What devices allow me to change my position and orientation? *Answer: Motors.*

(c) *Inference.* For any given position that a robot can reach, the robot arm can have a variety of different orientations. Can you describe an instance where a robot arm with 6 motors would have only one possible orientation? *Answer: Fully extended; bent back to the origin location; given a specific end effector position and orientation.*

(d) *Inference.* Can you describe an object where one orientation is no different from any other orientation? *Answer: Sphere.*

7. Waypoints

(a) *Ver Batim.* A robot has lifted an object and placed it into a container. If the robot wanted to pick up the object and place it back in the object's original location, what would it do? *Answer: Run the program in reverse.*

(b) *Integration.* When saving waypoints, what concept(s) is/are used? (A) Orientation and Position; (B) Kinematics; (C) Memory; (D) Motors; (E) Encoders. *Answer: A, C, and E.*

(c) *Inference.* If you wanted to have the end of the robot arm move through a series of waypoints as fast as possible, how would you have the arm move? *Answer: Without stopping and straight from point to point, in one motion.*

(d) *Inference.* Given this 2D maze, what is the minimum number of waypoints required to move through the maze from one entrance to the other? Assume you start at the entrance. *Answer: 10-12, the number of turns.*

References

[1] N. S. Selby, J. Ng, G. S. Stump, G. Westerman, C. Traweek, H. H. Asada, Teachbot evaluation question, 2019. URL: `https://www.youtube.com/watch?v=WisitfakeWw`.
